# Tbet Deficiency Causes T Helper Cell Dependent Airways Eosinophilia and Mucus Hypersecretion in Response to Rhinovirus Infection

**DOI:** 10.1371/journal.ppat.1005913

**Published:** 2016-09-28

**Authors:** Nicholas Glanville, Tamlyn J. Peel, Armin Schröder, Julia Aniscenko, Ross P. Walton, Susetta Finotto, Sebastian L. Johnston

**Affiliations:** 1 Airway Disease Infection Section, National Heart and Lung Institute, MRC & Asthma UK Centre in Allergic Mechanisms of Asthma, Imperial College London, London, United Kingdom; 2 Laboratory of Cellular and Molecular Lung Immunology, Department of Molecular Pneumology, Friedrich-Alexander-Universität Erlangen-Nürnberg, Erlangen, Germany; St. Jude Children's Research Hospital, UNITED STATES

## Abstract

Current understanding of adaptive immune, particularly T cell, responses to human rhinoviruses (RV) is limited. Memory T cells are thought to be of a primarily T helper 1 type, but both T helper 1 and T helper 2 memory cells have been described, and heightened T helper 2/ lessened T helper 1 responses have been associated with increased RV-induced asthma exacerbation severity. We examined the contribution of T helper 1 cells to RV-induced airways inflammation using mice deficient in the transcription factor T-Box Expressed In T Cells (Tbet), a critical controller of T helper 1 cell differentiation. Using flow cytometry we showed that Tbet deficient mice lacked the T helper 1 response of wild type mice and instead developed mixed T helper 2/T helper 17 responses to RV infection, evidenced by increased numbers of GATA binding protein 3 (GATA-3) and RAR-related orphan receptor gamma t (RORγt), and interleukin-13 and interleukin-17A expressing CD4+ T cells in the lung. Forkhead box P3 (FOXP3) and interleukin-10 expressing T cell numbers were unaffected. Tbet deficient mice also displayed deficiencies in lung Natural Killer, Natural Killer T cell and γδT cell responses, and serum neutralising antibody responses. Tbet deficient mice exhibited pronounced airways eosinophilia and mucus production in response to RV infection that, by utilising a CD4+ cell depleting antibody, were found to be T helper cell dependent. RV induction of T helper 2 and T helper 17 responses may therefore have an important role in directly driving features of allergic airways disease such as eosinophilia and mucus hypersecretion during asthma exacerbations.

## Author Summary

Rhinovirus infections cause the common cold and a high proportion of exacerbations of asthma and chronic obstructive airway disease. Little is understood of the roles of T cells in causing or limiting rhinovirus disease. Rhinovirus-specific memory cells are primarily of a T helper 1 type in healthy people, but rhinovirus-specific T helper 2 cells have been detected in blood of asthmatics. We examined the role of T helper 1 cells in rhinovirus disease using a mouse strain deficient in a key T helper 1 associated transcription factor. We show that mice deficient in T helper 1 cells instead develop a mixed T helper 2 and T helper 17 response to rhinovirus that was associated with a lack of neutralising antibody response and development of airway eosinophilia and mucus secretion, features of allergic asthmatic airways inflammation. Our study therefore provides new insight into how the nature of the rhinovirus-specific T helper cell response could influence rhinovirus-induced airways disease, especially in asthma, as well as generation of protective immunity.

## Introduction

Human rhinovirus (RV) infections cause the common cold and are associated with two thirds of asthma and one third of chronic obstructive pulmonary disease (COPD) exacerbations [1,2]. There are currently no specific licensed therapies or vaccines available for RV infections. Current understanding of adaptive immune responses to RV is very limited. Almost all studies have focused on the role of antibodies, showing that neutralising antibodies generated in response to infection can be protective against symptoms, but because of the antigenic heterogeneity amongst the >150 RVs people continue to suffer infections throughout life [3,4]. It is unknown what, if any, contribution conventional T cells make to virus control or to the severity of RV-induced colds. Similarly, whilst T helper cell responses have been associated with disease outcomes in asthma exacerbations, the contribution of RV-specific T cells has not specifically been studied. Studies of memory cells in humans have suggested that RV-specific T cells in tonsil and peripheral blood are primarily CD4+ helper cells which express the Th1 cytokine interferon (IFN)-γ upon re-stimulation with RV [5–7]. However, production of the Th2 cytokines interleukin (IL)-4, IL-5 and IL-13 by RV-specific memory cells has also been described and IL-4 and IL-13 have been detected in supernatants of RV-exposed peripheral blood mononuclear cells (PBMC) from asthmatics, suggesting that Th2 responses to RV develop in some individuals [5,6,8]. RV infection in fact induces both Th1 promoting factors such as C-X-C motif ligand (CXCL)10 and IL-12, and Th2 promoting factors C-C motif ligand (CCL)17, CCL22, IL-33 and IL-25 *in vivo* and/or *in vitro* [9–13]. RVs therefore appear to have the capacity to induce both Th1 and Th2 orientated responses. For other respiratory viruses such as respiratory syncytial virus (RSV) for which T cell immunity is better understood, T cells have complex roles, both aiding virus clearance but also causing immunopathology, with alterations in the balance between type 1 and type 2 T cell responses having been linked to severity of virus associated immunopathology [14]. The limited available evidence for RV suggests that Th1 responses are desirable because we have shown in the mouse that enhancing the memory Th1 response by means of vaccination is associated with enhanced neutralising antibody responses and in humans, that greater Th1 and lessened Th2 number following polyclonal stimulation are associated with improved disease outcome during experimental RV-induced asthma exacerbations[15,16]. It remains to be fully established however what type of response is most desirable in terms of limiting virus replication and potentially reducing disease, and what exactly the implications of an aberrant T helper response might be.

We therefore addressed the question of whether Th1 responses to RV are required for virus control and what influence they have on other aspects of airway disease by employing the mouse model of infection and mice deficient in T-Box Expressed In T Cells (Tbet), a transcription factor which promotes Th1 and suppresses both Th2 and Th17 differentiation. We showed that whilst wild type mice developed Th1 responses to RV, Tbet deficient mice instead developed a mixed Th2/Th17 response. This altered response was associated with T helper cell-dependent airways eosinophilia and mucus secretion reminiscent of asthmatic airways inflammation. RV-specific Th2/Th17 responses, if developed in asthmatics as has been proposed, could therefore have an important role in enhancing disease during RV-induced disease exacerbations.

## Results

### Tbet deficient mice develop Th2 and Th17 responses to RV

**Fig 1:**
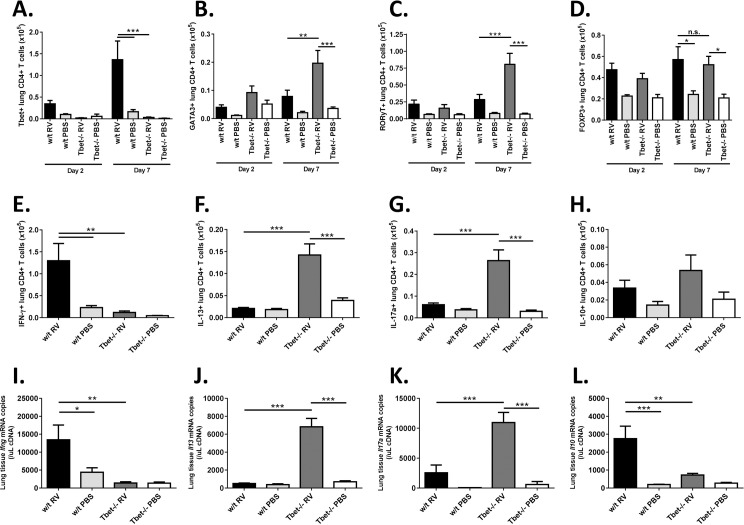
Helper T cell responses in Tbet deficient and wild type mice. Wild type and Tbet-/- mice were infected intranasally with RV1B or sham infected with PBS. (A-D) Intranuclear flow cytometry staining for transcription factors Tbet (A), GATA-3 (B), RORγt (C) and FOXP3 (D) in CD3+CD4+ lung T cells, 2 and 7 days post-infection. (E-H) Intracellular flow cytometry staining for cytokines IFN-γ (E), IL-13 (F), IL-17A (G) and IL-10 (H) in lung CD3+CD4+ cells stimulated with PMA and ionomycin, on day 7 post-infection. (I-L) RNA was extracted from lung tissue harvested on day 7 post-infection and expression of IFN-γ (I), IL-13 (J), IL-17a (K) and IL-10 (L) mRNA was quantified by Taqman qPCR. n = 8–9 mice/group.***p<0.001, **p<0.01, *p<0.05, n.s. not significant.

To confirm that Tbet-/- mice lacked Tbet expression in helper T cells following RV infection, we performed flow cytometry staining of lung T cells harvested from Tbet-/- and wild type (w/t) control mice 2 and 7 days after infection. RV infected w/t mice displayed an increase in the number of CD3+CD4+ T cells expressing Tbet compared to phosphate buffered saline (PBS) challenged w/t animals on day 7 post-infection (Fig 1A). As expected, Tbet-/- mice lacked Tbet expressing CD3+CD4+ cells. Instead, RV infected Tbet-/- mice had increases in lung CD3+CD4+ T cells expressing both GATA binding protein 3 (GATA-3) (Fig 1B), the Th2 associated transcription factor, and RAR-related orphan receptor gamma t (RORγt) (Fig 1C), the Th17 associated transcription factor, compared to both RV infected w/t and PBS challenged Tbet-/- mice on day 7 post-infection. We also enumerated forkhead box P3 (FOXP3) expressing CD4+ T cells as a measure of Treg responses. The number of FOXP3 expressing lung CD3+CD4+ T cells was increased at 7 days post-infection in both w/t and Tbet-/- mice, but was not different between the 2 mouse strains (Fig 1D).

To confirm this T helper phenotype, we performed intracellular cytokine staining (ICS) for Th1 (IFN-γ), Th2 (IL-13), Th17 (IL-17A) and Treg (IL-10) associated cytokines in polyclonally stimulated lung leukocytes on day 7 post-infection. Consistent with the transcription factor staining, IFN-γ expressing CD4+ T cell number was increased in RV infected vs. PBS challenged w/t, but not Tbet-/- mice (Fig 1E), whereas IL-13 (Fig 1F) and IL-17A (Fig 1G) expressing cell number was increased in RV infected Tbet-/- mice compared to both RV infected w/t mice and PBS challenged Tbet-/- mice. IL-10 expressing cell number was not significantly increased following RV infection in either mouse strain (Fig 1H). Differences in Th1, Th2 and Th17 cytokine expression between RV infected w/t and Tbet-/- mice were supported by changes in lung tissue cytokine mRNA expression (Fig 1I–1K). *Il10* mRNA levels were increased in RV infected w/t mice compared to PBS challenged w/t mice and compared to RV infected Tbet-/- mice (Fig 1L). These findings are consistent with a mixed Th2/Th17 helper T cell response to RV in Th1 deficient Tbet-/- mice.

**Fig 2:**
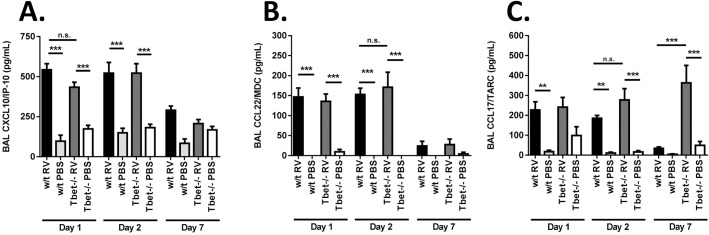
Airway chemokine expression in RV infected Tbet deficient and wild type mice. Wild type and Tbet-/- mice were infected intranasally with RV1B or sham infected with PBS. On days 1, 2 and 7 post-infection lungs were lavaged and chemokines CXCL10/IP-10 (A), CCL22/MDC (B) and CCL17/TARC (C) in lavage supernatants were measured by ELISA. n = 7–8 mice/group. ***p<0.001, **p<0.01, *p<0.05, n.s. not significant.

In addition to T cell transcription factor and cytokine expression, we also measured airway T cell chemoattractant levels in Tbet-/- vs. w/t mice. On days 1 and 2 post-infection the Th1 associated C-X-C motif chemokine receptor (CXCR)3 ligand CXCL10/IP-10 (Fig 2A) and the Th2 associated C-C motif chemokine receptor (CCR)4 ligands CCL22/MDC (Fig 2B) and CCL17/TARC (Fig 2C) in bronchoalveolar lavage (BAL) were similarly induced in RV infected Tbet-/- and w/t mice compared to the respective PBS challenged controls. Levels of CCL17/TARC in BAL were however significantly elevated in RV infected Tbet-/- vs. w/t control mice on day 7 post-infection.

### Tbet deficiency alters antibody responses to RV

**Fig 3:**
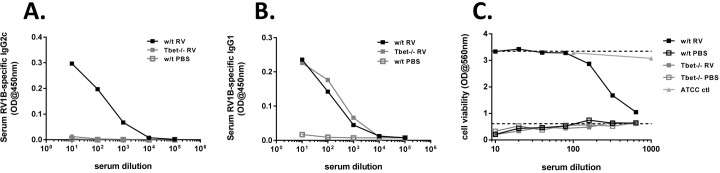
RV-specific antibody responses in Tbet deficient and wild type mice. Wild type and Tbet-/- mice were infected intranasally with RV1B or sham infected with PBS. Blood was collected 14 days after infection. (A&B) RV1B-binding IgG2c (A) and IgG1 (B) in sera was measured by ELISA. (C) Neutralisation of RV1B infection of Ohio HeLa cells by pooled sera assessed by crystal violet cell viability staining. ATCC ctl: control reference guinea pig anti-sera. Top dashed line in C, uninfected cells control. Bottom dashed line in C, RV infected cells control. Data represent results from 5–6 pooled sera per treatment group in a single experiment, representative of 3 independent experiments.

Changes in T cell response might be expected to have effects on antibody responses due to the roles of T cell cytokines on antibody class switching. Consistent with this, we found that RV-specific serum immunoglobulin (Ig)G2c, a Th1 associated antibody isotype, was detectable in RV infected w/t mice but completely absent in RV infected Tbet-/- mice (Fig 3A). Despite the increase in Th2 responses however, levels of RV-specific IgG1, a Th2 associated antibody isotype, were comparable in Tbet deficient and w/t mice following infection (Fig 3B). These changes in RV binding antibodies in Tbet-/- mice were associated with a complete lack of serum neutralising antibody in RV infected Tbet-/- mice on day 14 post-infection, in contrast to RV infected w/t mice (Fig 3C).

### Tbet deficiency causes airway eosinophilia and mucus hypersecretion

**Fig 4:**
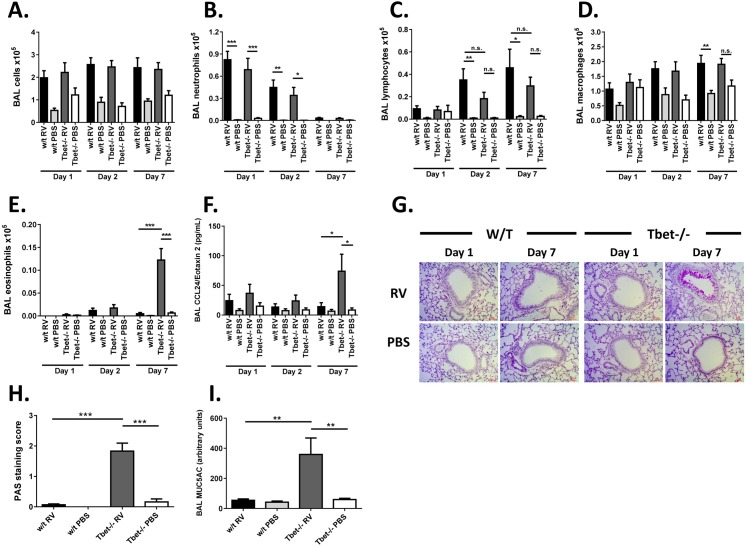
Airways inflammation in RV infected Tbet deficient and wild type mice. Wild type and Tbet-/- mice were infected intranasally with RV1B or sham infected with PBS. (A-E) Lungs were lavaged on days 1, 2 and 7 post-challenge. Total cells were counted (A), and neutrophils (B), lymphocytes (C), macrophages (D) and eosinophils (E) were enumerated by differential counting of cytospin slides. (F) CCL24/eotaxin2 levels in BAL supernatant as measured by ELISA. (G) Representative images and (H) scores for PAS mucin staining in paraffin wax embedded lungs harvested on day 7 post-infection. (I) Levels of mucin protein MUC5AC in BAL supernatants on day 7 post-challenge as measured by ELISA. Scale bars 50μm. n = 7–8 mice/group. ***p<0.001, **p<0.01, *p<0.05, n.s. not significant.

We next determined what effect the altered T helper cell response to RV in Tbet-/- mice had on other aspects of airways inflammation. The cellular airways response to RV in the mouse is typically characterised by early neutrophilia followed by an increase in lymphocyte number peaking around days 4–7 post-infection [17]. Total numbers of cells in BAL were not different between RV infected Tbet-/- and RV infected w/t mice at any timepoint studied (Fig 4A). Similarly, the neutrophil response was unaltered by Tbet deficiency, with increases in neutrophils in BAL evident in both RV infected w/t and RV infected Tbet-/- mice on days 1 and 2 post-infection compared to their respective PBS challenged controls (Fig 4B). Lymphocyte number in BAL was significantly increased in RV vs. PBS challenged w/t, but not Tbet-/- mice on days 2 and 7 post-challenge (Fig 4C). Lymphocyte numbers however did not significantly differ between RV infected Tbet-/- and w/t groups. Macrophage number in BAL was unaffected by Tbet deficiency (Fig 4D). RV infected Tbet-/- mice did however develop significant eosinophilia in the airways at 7 days post-infection, a finding that was not observed in any other treatment group (Fig 4E). Similarly to the association of increased CCR4 ligand levels with Th2 cells, the eosinophilic airways response to RV in Tbet-/- mice was associated with an increase in the eosinophil chemoattractant CCL24/Eotaxin 2 in the airways compared to PBS challenged Tbet-/- and RV infected w/t mice on day 7 post-infection (Fig 4F). Mucin production is a characteristic feature of RV infected epithelial cells and mucus production represents a symptom of RV infection and asthma in humans [18]. We therefore measured airway mucin producing cells via Periodic Acid Schiff (PAS) staining of lung sections and mucin 5AC (MUC5AC) protein levels in BAL. There was no evidence of PAS positive cells in the airways of any treatment group on day 1 post-challenge, but by day 7 post-infection there was strong PAS staining in RV infected Tbet-/- mice that was absent in infected w/t mouse lungs (Fig 4G and 4H). This finding was supported by a significant increase in BAL MUC5AC protein levels in RV infected Tbet-/- vs. w/t mice 7 days after infection (Fig 4I). Low powered images of PAS stained lungs are shown in S1 FIg.

### Tbet deficiency is not associated with impaired virus control

**Fig 5:**
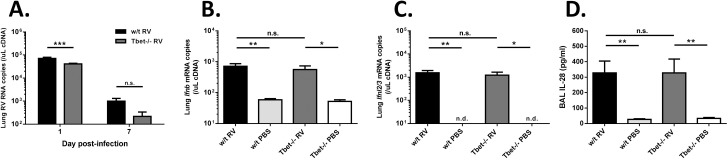
Lung virus loads and interferon responses in Tbet deficient and wild type mice. Wild type and Tbet-/- mice were infected intranasally with RV1B or sham infected with PBS. Lung tissue was harvested on days 1, and 7 post-challenge, RNA extracted and RNA levels were quantified by Taqman qPCR. (A) Lung viral RNA on days 1 and 7 post-infection. (B) *Ifnb* and (C) *ifnl2/3* mRNA levels on day 1 post-infection. (D) Lungs were lavaged on day 1 post-infection and IL-28 levels measured by ELISA. n = 8 mice/group. ***p<0.001, **p<0.01, *p<0.05, n.s. not significant.

Given both our understanding of the role of T cell subsets in other respiratory virus infections and the fact that the limited studies of RV antigen-specific memory T cells in humans show a strong Th1 bias, we hypothesised that Th1 cells might directly or indirectly have an antiviral role. Measurement of viral RNA in lung tissue however showed that lung virus levels on both day 1 and day 7 post-challenge were not increased by Tbet deficiency (Fig 5A). In fact, RV RNA levels were significantly lower on day 1 post-infection in Tbet-/- vs. w/t mice. This difference in lung virus load was not due to differences in induction of innate antiviral mediators, because *Ifnb* and *Ifnl2/3* (interferon-λ2/3) mRNA levels in lung tissue, and interferon-λ protein levels in BAL were no different in RV infected Tbet-/- and w/t mice (Fig 5B–5D).

### Tbet deficiency also causes deficiencies in innate cellular responses to RV

**Fig 6:**
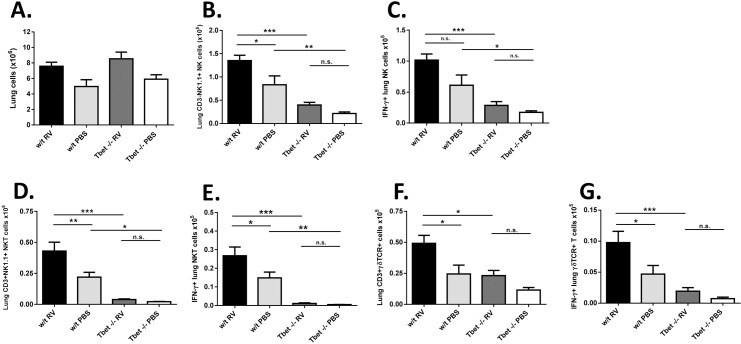
Effect of Tbet deficiency on innate cellular responses to RV. Wild type and Tbet-/- mice were infected intranasally with RV1B or sham infected with PBS. On day 2 post-challenge lungs were harvested and flow cytometry staining was performed. For intracellular cytokine staining, lung cells were stimulated with PMA and ionomycin. (A) Total numbers of lung cells. (B-G) Numbers of CD3+NK1.1- Natural Killer (NK) cells (B), IFN-γ expressing NK cells (C), CD3+NK1.1+ NKT cells (D), IFN-γ expressing NKT cells (E), CD3+γδTCR+ T cells (F) and IFN-γ expressing CD3+γδTCR+T cells (G). n = 7–8 mice/group. ***p<0.001, **p<0.01, *p<0.05, n.s. not significant.

Tbet is also expressed in a number of other cells of the immune system, most notably Natural Killer (NK) cells and NK T cells. We therefore measured NK and NKT, as well as γδT cell responses to RV infection to determine if deficiencies in these innate cells were present that could contribute to the inflammatory phenotype found in Tbet-/- mice. Innate responses were measured around their peak at 2 days after infection [19,20]. PBS challenged Tbet-/- mice had similar total lung cell counts (Fig 6A) but fewer NK (Fig 6B), IFN-γ expressing NK (Fig 6C), NKT (Fig 6D) and IFN-γ expressing NKT cells (Fig 6E) compared to PBS challenged w/t mice, indicating a basal deficiency in these cells. Infection did not overcome this deficiency because RV infection significantly increased NK, NKT and IFN-γ producing NK and NKT number in the lungs in w/t mice, but not Tbet-/- mice. Lung γδT cell (Fig 6F) and IFN-γ producing γδT cell (Fig 6G) number was not significantly different in PBS challenged Tbet-/- vs. w/t mice, but again failed to increase following infection in Tbet-/- mice, such that total and IFN-γ+ γδT cell number was higher in RV infected w/t vs. RV infected Tbet-/- mice.

### Airways eosinophilia and mucus secretion in Tbet deficient mice are T cell dependent

**Fig 7:**
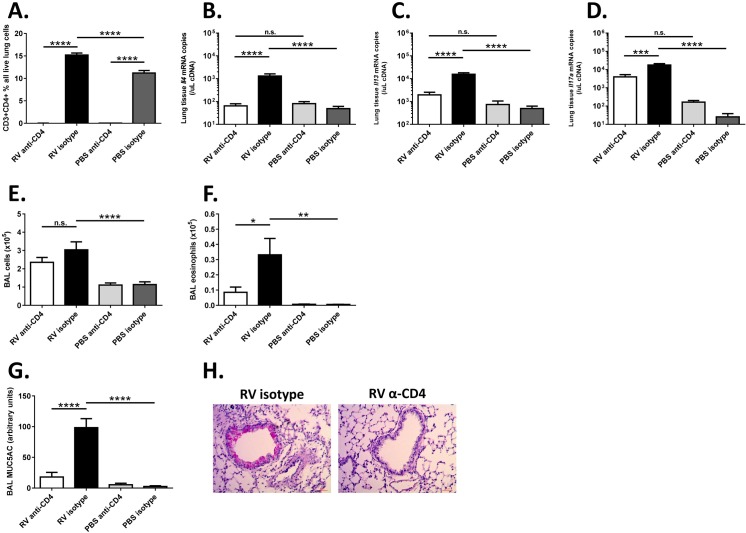
Asthma-like airways inflammation is CD4+ T cell dependent. Tbet-/- mice were infected intranasally with RV1B or sham infected with PBS. In addition, mice were systemically depleted of CD4 expressing cells (anti-CD4), or treated with isotype control antibody (isotype) 3hrs prior to infection. Tissues were harvested at 7 days post-challenge. (A) Lung flow cytometry staining for CD3+CD4+ T cells. (B-D) Levels of cytokines IL-4 (B), IL-13 (C) and IL-17a (D) in lung tissue measured by Taqman qPCR. (E) Total BAL cell counts and (F) eosinophil counts in BAL measured by cytospin assay. (G) MUC5AC levels in BAL measured by ELISA. (H) Representative PAS staining for mucus in lung tissue sections. Scale bars 50μm. n = 12–15 mice/group (mice for which CD4+ cell depletion was not successful were excluded from all analyses (n = 5 of 30)). ***p<0.001, **p<0.01, *p<0.05, n.s. not significant.

Given that Tbet-/- mice displayed deficiencies in innate cellular responses to RV, we next systemically depleted CD4+ T cells in Tbet deficient mice to determine if the altered inflammatory phenotype in Tbet-/- mice was dependent upon T helper cells. Administration of a single dose of a well characterised CD4+ cell depleting monoclonal antibody on day 0, 3 hours prior to infection, led to a complete absence of detectable CD4+ T cells in the lungs of both RV and PBS challenged Tbet-/- mice at 7 days post-challenge (Fig 7A). Whilst T helper cells could obviously not be measured in antibody treated mice, an absence of induction of *Il4* (Fig 7B), *Il13* (Fig 7C) and *Il17a* (Fig 7D) mRNAs in the lung following infection supported an absence of Th2 and Th17 responses in CD4+ cell depleted mice.

Total BAL cell number (Fig 7E) was not significantly affected by CD4+ cell depletion. BAL eosinophils (Fig 7F) were however significantly reduced in RV-infected anti-CD4 vs. RV-infected isotype control treated mice. As shown in Fig 4, RV-infected Tbet-/- mice had high levels of MUC5AC in BAL (Fig 7G) and large amounts of PAS staining in the airway epithelium (Fig 7H) 7 days after infection. Both mucin protein induction and PAS staining were almost completely abolished in RV infected mice treated with anti-CD4 antibody.

## Discussion

We utilised a Tbet deficient mouse strain to examine the role of Th1 cell responses in RV infection. We found that in the absence of Tbet, Th2 and Th17 responses developed in the airways and that this altered T cell response was associated with an impaired antibody response and drove an inflammatory phenotype characterised by airways eosinophilia and enhanced mucus production.

The Th2 and Th17 responses that developed in the RV infected mouse lung can be explained by the well-established role of Tbet in both facilitating Th1 differentiation and at the same time supressing Th2 and Th17 differentiation. Tbet directly induces IFN-γ gene expression and therefore effector functions of Th1 cells, as well as up-regulating expression of the Th1 cell homing receptor CXCR3 [21,22]. Tbet also inhibits Th2 differentiation by repressing expression of the Th2 differentiation promoting transcription factor GATA-3, binding of the *IL4* silencer and suppression of *IL5* and *IL13* expression via sequestration of GATA-3 away from Th2 genes[22–25]. Tbet also inhibits Th17 differentiation via a number of possible mechanisms such as blocking Runt-related transcription factor (RUNX)1 activation of *Rorc*, the gene encoding the Th17 associated transcription factor RORγt [26]. Tbet is also expressed in some regulatory T cells (Treg), although its function in these cells is not well understood [27]. We saw an increase in FOXP3+ CD4+ T cells in the lungs of RV infected mice, but neither this nor the proportion of CD4+ T cells expressing IL-10 was affected by Tbet deficiency. Tbet deficient mice did however display a reduction in lung *Il10* mRNA levels after infection, suggesting a possible deficiency in IL-10 production from a non-helper T cell source.

RV induces a number of factors in mice that are associated with Th2 responses, such as IL-25 and CCR4 ligands [11,13]. In RV infected Tbet deficient mice we observed an increase in airway levels of the chemokine CCL17, a ligand for CCR4 which is expressed on Th2 cells, on day 7 post-infection. This suggests that Th2 trafficking in addition to differentiation might be enhanced in Tbet deficient mice. Given that CCL17 was expressed in RV infected wild type mice on days 1 and 2 post-infection, but did not result in Th2 responses, it would appear however that a lack of Tbet mediated suppression of Th2 differentiation in lymphoid tissue must precede chemokine expression for a Th2 response to occur. We also did not show that increased CCL17 expression in RV infected Tbet deficient mice preceded Th2 recruitment, as one might expect, due to a lack of intermediate timepoints between days 2 and 7 to our studies. This is true also of the association of increased CCL24 levels in the airways of RV infected Tbet deficient mice with airways eosinophilia and further work is therefore required to determine whether chemokines play a role in the Th2 and eosinophil responses observed in Tbet deficient mice.

Th2 inducing factors are also expressed following RV infection in otherwise healthy humans without there being measureable Th2 responses. RV infection of asthmatics does however enhance airway Th2 responses and it is assumed in this case that RV infection is primarily enhancing allergen-specific Th2 cell responses in the asthmatic airways [12]. However, to date nobody has assessed the antigen specificity of Th2 cells during RV-induced asthma exacerbations and some investigators have described RV-specific Th2 memory based on analysis of peripheral blood cells [5,8]. If RV-specific Th2 responses occur in the human airways during infection, then our finding that RV-specific Th2 or Th17 cells can drive airways eosinophilia and mucus hypersecretion could have important implications in terms of understanding RV-induced asthma disease mechanisms. Th1 (Tbet) deficiency also had significant effects on antibody responses to RV, causing an absence of IgG2c and no difference or a small increase in the IgG1 response. This is very much consistent with the roles of T cell derived cytokines in B cell class switching, whereby IFN-γ is associated with IgG2a switching (IgG2c in C57BL/6 mice is analogous to IgG2a in humans and other mouse strains) and Th2 cytokines IL-4 and IL-5 with switching to IgG1. Tbet is also expressed in B cells where it has been associated with class switching to IgG2a, even in a T cell independent system [28,29]. More interesting however is the finding that Tbet deficiency completely abrogated the neutralising antibody response, suggesting that Tbet/Th1 cells are absolutely required for neutralising antibody generation and that neutralising antibodies might be exclusively IgG2a. This is consistent with a previous finding that TLR-9 agonist administration enhances neutralising antibody responses to RV in mice, but no other study has examined antibody isotypes or type of T cell help required for generation of neutralising antibody to RV [30].

We only measured lung virus loads in terms of viral RNA in lung tissue, not viable virus in the airways, but the lack of neutralising antibody response did not increase virus loads in the lung by this measure and in fact, viral RNA levels in the lung were greater in Tbet-/- mice 24hrs after infection. The reason for the higher viral RNA levels in Tbet-/- mice soon after infection are not known, but it is perhaps not surprising that lung virus loads were not increased despite an absence of neutralising antibody given that neutralising antibodies are not measureable in the mouse until at least a week after infection, when virus has largely been cleared from the lung. In humans however virus tends to persist longer, with viral RNA levels peaking in the nose around 3 to 4 days after infection and persisting until at least 10 days post-infection in experimental infection studies [12,16]. Diminished neutralising antibody responses could therefore have negative consequences in terms of virus clearance in man. This is also an important consideration in terms of generation of protective immunity in humans, suggesting that asthmatics with greater Th2 responses might be impaired in their ability to develop protective neutralising antibody. In terms of future vaccine design, our findings suggest that Th1 promoting adjuvants will likely be required for developing protective antibody responses after vaccination.

An array of immune cells other than helper T cells also express Tbet, including dendritic cells (DC), CD8+ T cells, Innate Lymphoid Cells, Natural Killer cells, Natural Killer T cells and γδT cells [31]. We found NK, NKT and γδT cell responses to be deficient in the lungs of Tbet-/- mice following infection. NK and NKT cells were in fact deficient in the lungs even without virus challenge, consistent with the role of Tbet, along with Eomes, in their development and survival [32,33].

With the exception perhaps of DC, all of the cell types listed above have effector functions which can overlap to a lesser or greater degree with Th1 cells. We have in fact shown previously that NK cells are an early source of IFN-γ in the mouse RV infection model, with a possible role in virus control [19]. We therefore depleted CD4+ cells in Tbet-/- deficient mice to show that the inflammatory phenotype exhibited was dependent on the aberrant T helper cell response. Depletion of CD4+ T cells resulted in a near complete reversal of RV induced eosinophilia and mucus production, suggesting that these features were indeed dependent on CD4+ T cells. Though this finding shows a CD4+ T cell dependence, it does not establish that CD4+ T cells were sufficient to drive this inflammatory phenotype, and a number of other cells may contribute. For example, Tbet deficiency in DCs is associated with an impaired ability to prime Th1 responses [34]. Nevertheless, the T helper cell dependence is consistent with the many reports that Th2 and Th17 associated cytokines can facilitate airway mucus cell metaplasia and/or eosinophilia [35–37]. More specifically, Tbet deficient T cells have been shown to drive airway IL-4 production and hyperreactivity in an asthma model and both IL-13 and IL-17A have been shown to be able to drive allergic airways disease in Tbet deficient mice [38–40].

In summary, we have shown that deficiencies in Th1 responses led to the development of Th2 and Th17 responses to RV. These altered T helper responses were associated with development of some features of asthmatic airways inflammation in mice and may therefore contribute to asthma exacerbations in humans. New treatments which enhance Th1 responses or replace deficient Th1 promoting/Th2 supressing factors in RV infections, such as the recombinant type I/III interferons which are currently in development, could therefore be beneficial in limiting such Th2 and Th17 mediated RV disease.

## Materials and Methods

### Ethics statement

All of the animal studies described were carried out under the authority of the UK Home Office (Animals (Scientific Procedures) Act 1986) project licence number PPL 70/7234. Intranasal dosing was performed under light, transient anaesthesia with isoflurane. Animals were culled by overdose of pentobarbitone.

### Mice

Homozygous Tbet knockout (Tbet-/-) (B6.129S6-Tbx21tm1Glm/J) and wild type (w/t), female C57BL/6J mice were obtained from Charles River Laboratories UK and were housed in individually ventilated cages. Mice were 6–8 weeks old at the commencement of studies. All studies were conducted according to the UK Animals (Scientific Procedures) Act 1986 under the authority of project licence number PPL 70/7234.

Mice were infected with 5x10^6^ TCID_50_ RV1B, in 50uL volume, intranasally under anaesthesia with isoflurane. CD4 expressing cells were depleted in the indicated studies by intraperitoneal administration of 0.2mg anti-mouse CD4 antibody clone GK1.5 (BioXCell, New Hampshire, USA) in 150μL PBS, 3hrs prior to infection. Control mice were administered 0.2mg isotype control rat IgG2b clone LTF-2 (BioXCell). Depletion efficacy was assessed by flow cytometry staining of lung cells. Depletion was effective by 24hrs post-administration and was assessed in the studies shown on day 7 post-infection. Where depletion was not successful (<99% reduction in lung CD3+CD4+ cells; n = 3 of 15 RV-anti-CD4, n = 3 of 15 PBS anti-CD4), mice were excluded from all other analyses.

### Tissue processing

Lung tissue was processed for flow cytometry by homogenisation with the gentleMACS tissue dissociator (Miltenyi Biotech GmbH, Germany) and digestion in medium containing 1mg/mL collagenase A from *Clostridium histolyticum* (Roche Diagnostics, West Sussex, UK) and 80 units/mL Deoxyribonuclease I from bovine pancreas (Sigma-Aldrich, Dorset, UK). Red cells were lysed with ACK buffer.

Lungs were lavaged 4 times via the trachea with 1.5mL of PBS containing 55mM EDTA. Lavage cells were separated by centrifugation and spun onto cytospin slides for subsequent differential staining. Blood was collected from the carotid arteries and serum separated by centrifugation using Microtainer SST tubes (BD Biosciences, Oxford, UK).

The apical lobe of the right lung was harvested from mice after lavage was performed and was stored in RNAlater buffer (Qiagen, Manchester, UK) for subsequent RNA analysis. RNA was extracted using the RNeasy mini kit (Qiagen) and cDNA generated using the Omniscript RT kit (Qiagen) and random primers.

### RV propagation

RV serotype 1B was propagated in H1 HeLa cells (American Type Tissue Culture Collection (ATCC) ref CRL-1958) and purified using previously described methods [17]. Virus was titrated on Ohio HeLa cells (European Collection of Authenticated Cell Cultures) and TCID_50_ was calculated using the Spearman-Karber formula. Purified, uninfected H1 HeLa cell lysate was generated in the same manner for use as a control in virus-specific antibody assays.

### Flow cytometry staining

Flow cytometry was performed on lung leukocytes obtained by collagenase digestion as described. For intracellular cytokine staining, cells were stimulated for 4hrs in medium containing 50ng/mL Ionomycin, 500ng/mL Phorbol myristate acetate (PMA) (both Sigma Aldrich) and golgi transport inhibitor (Golgi Stop; BD Biosciences) before staining. In brief, 1x10^6^ cells were stained with live/dead fixable dead cell stain (Life Technologies, Paisley, UK), Fc receptors were blocked with anti-CD16/CD32 (BD Biosciences) and cells were then incubated with fluorochrome-conjugated antibody cocktails. Antibodies used were anti-CD4 clone RM4-5 (BD Biosciences), anti-CD3 clone eBio500A2 (eBioscience, San Diego, CA, USA), anti-NK1.1 clone PK136, anti-γδTCR clone GL3, anti-IL-17a clone TC11-18H10 (all BD Biosciences), anti-IFN-γ clone XMG1.2 (Biolegend, San Diego, CA, USA), anti-IL-13 clone eBio13A, anti-IL-10 clone JES5-16E3 (both ebioscience), anti-Tbet clone 04–46 (BD Bioscience), anti-FOXP3 clone FJK-16s, anti-RORγT clone B2D (both eBioscience) and anti-GATA3 clone L50-823 (BD Bioscience). Intracellular staining was performed using the Foxp3/Transcription Factor Staining Buffer Set (eBioscience). Flow cytometry data was acquired with a BD LSR Fortessa cytometer and analysed using FlowJo software vX0.6 (FLOJO LLC, OR, USA). T helper cells were defined as live/dead marker negative, single cells, forward scatter/side scatter low, CD3 vs. CD4 double positive. CD4 was further plotted against intracellular transcription factors or cytokines for Fig 1.

### ELISA and neutralisation assays

Cytokine and chemokine proteins in BAL were assayed using protocols and reagents from Duoset ELISA kits (R&D systems, Minneapolis, MN, USA). Assay ranges were as follows. CXCL10 62.5–4,000 pg/mL, CCL22 7.8–500 pg/mL, CCL17 31.2–2,000 pg/mL, CCL24 15.6–1,000 pg/mL.

RV-specific IgGs were measured using in-house assays. 96 well plates were coated with purified RV1B inoculum or HeLa lysate control overnight and blocked with PBS 5% skimmed milk. Treatment group sera were pooled and diluted in PBS 5% milk before being added to plates. Detection antibodies were biotinylated rat anti-mouse IgG1 (clone A85-1) and IgG2a/c (clone R19-15) (both BD biosciences) diluted in PBS 1% BSA. Plates were developed by incubation with streptavidin- horseradish peroxidase and TMB substrate (both Sigma Aldrich). Values generated by antibody binding to a HeLa lysate control wells were subtracted from that of RV1B coated wells during analysis.

MUC5AC was measured using an in-house, semi-quantitative, direct ELISA assay. Plates were coated with BAL fluid, diluted 1 in 10 in PBS, or cell culture supernatant standard and allowed to dry. After washing and blocking, biotinylated detection antibody (anti-MUC5AC clone 45M1, Fisher Scientific, Loughborough, UK) was applied. Plates were developed by standard methods.

Serum neutralisation of RV was measured in Ohio HeLa cells. Pooled sera were heat inactivated at 56°C for 45minutes, then diluted and incubated with RV1B before addition of HeLa cells. A reference anti-RV1B anti-sera (American Type Culture Collection) was used as a positive control. After 48-72hrs at 37°C cytopathic effect (CPE) was measured by staining with crystal violet. After drying, crystal violet was re-dissolved in 1% SDS and absorbance was measured at 560nm.

### Quantitative PCR

Cytokine/interferon mRNAs and viral RNA in lung tissue were measured by Taqman qPCR using Quantitect probe PCR mastermix (Qiagen) and were normalised to *18S* ribosomal RNA. Primer and probe sequences for *Il13* [41], RV, *Ifnβ*, *Ifnl2/3* and *18s* rRNA [42] have been described previously. *Il10* mRNA was measured using a commercial Taqman gene expression assay (Mm00439616_m1, Life Technologies). Primer sequences (5’-3’) for other assays are as follows. *Ifng* forward TCA AGT GGC ATA GAT GTG GAA GAA, *Ifng* reverse TGG CTC TGC AGG ATT TTC ATG, *Ifng* probe FAM-TCA CCA TCC TTT TGC CAG TT-TAMRA; *Il4* forward ACA GGA GAA GGG ACG CCA T, *Il4* reverse GAA GCC CTA CAG ACG AGC TCA, *Il4* probe FAM-TCC TCA CAG CAA CGA AGA-TAMRA; *Il17a* forward TCAGACTACCTCAACCGTTCCA, *Il17a* reverse AGCTTCCCAGATCACAGAGGG, *Il17a* probe TCACCCTGGACTCTCCACCGCA.

### Histology

For histology, lungs were inflated with 4% paraformaldehyde, removed from the thorax and immersed in 4% paraformaldehyde. Lungs were paraffin wax embedded, cut into 5μM sections and stained with periodic acid-Schiff (PAS) to detect mucus producing cells. PAS staining was scored using a previously described system adapted from Semitekolou et al [43], in which 0 = <5% of airway PAS positive, 1 = 5–25% of airway PAS positive, 2 = 25–50% of airway PAS positive, 3 = 50–75% of airway PAS positive, 4 = >75% of airway PAS positive. 15 airways were counted per section and averaged. Scoring was performed blind. Images were acquired using a Zeiss AxioScope A1 microscope with A-plan 10x (0.25 aperture) (Figs 4 and 7) or 2.5x (S1 Fig) objective, Zeiss Axiocam ERc5s camera and Zen2012 Blue Edition version 1.1.1.0 software (Carl Zeiss Ltd, Cambridge, UK).

### Statistical analysis

Graphical data is expressed as mean +/- SEM. Differences were assessed by 1 or 2-way ANOVA followed by Bonferroni post-test to pinpoint specific differences. Differences were considered significant if p<0.05. Analyses were performed using Prism software v6 (GraphPad Software, La Jolla, CA, USA).

## Supporting Information

S1 Fig PAS stained lung sections in RV infected Tbet deficient and wild type mice. Wild type and Tbet-/- mice were infected intranasally with RV1B or sham infected with PBS. Representative low powered images for PAS mucin staining in paraffin wax embedded lungs harvested on day 7 post-infection. Scale bars 20μm.
(PDF)
