# Supplementary figures and images for: Tbet Deficiency Causes T Helper Cell Dependent Airways Eosinophilia and Mucus Hypersecretion in Response to Rhinovirus Infection
Source: PLoS Pathog. 2016 Sep 28;12(9):e1005913. doi: 10.1371/journal.ppat.1005913 (PMC5040449; doi:10.1371/journal.ppat.1005913)

## S1 Figure

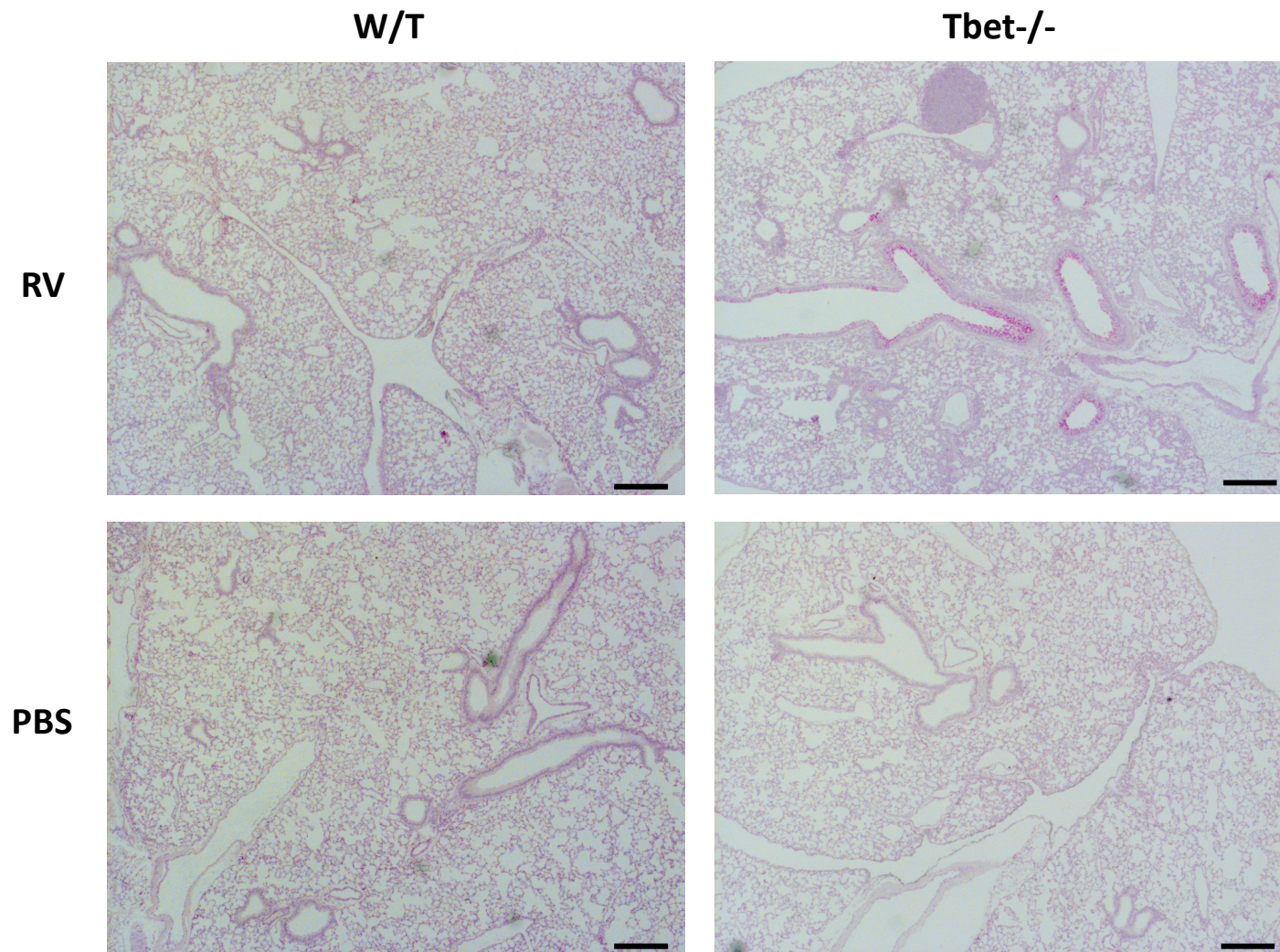

Supplement: S1 Fig — Wild type and Tbet-/- mice were infected intranasally with RV1B or sham infected with PBS. Representative low powered images for PAS mucin staining in paraffin wax embedded lungs harvested on day 7 post-infection. Scale bars 20μm. (PDF) [file ppat.1005913.s001.pdf]
